# Internal jugular vein aneurysm: A case report

**DOI:** 10.1097/MD.0000000000009588

**Published:** 2018-01-12

**Authors:** Abdulrahman M. Nasiri, Nora Rayes, Khaled A. Bakarman

**Affiliations:** a College of Medicine, Al Imam Mohammad Ibn Saud Islamic University; b College of Medicine, Princess Nora bint Abdul Rahman University; c Department of General Surgery, Vascular Surgery Division, Prince Sultan Military Medical City, Riyadh, Saudi Arabia.

**Keywords:** aneurysm, arterial aneurysm, dilatation, internal jugular vein, venous

## Abstract

**Introduction:**

Aneurysm is a localized dilatation of an artery of at least 1.5 times the normal diameter that occurs when part of an artery wall weakens or is injured, allowing it to widen abnormally. In practice, an arterial aneurysm is more common in comparison to a venous aneurysm. Because of the rare incidence of venous aneurysms, treatment guidelines are not clearly established and thus treatment strategies vary. This is a case of a 57-year-old Saudi woman, with no significant medical history, who presented to Prince Sultan Military Hospital complaining of swelling in the right side of the neck that started 3 years ago. The patient reported that the swelling enlarged with coughing and straining, but there was no pain, change in skin color, dysphagia, change in voice, neurological defect, shortness of breath, history of any trauma to the neck, surgical intervention, or any lump. The condition can be diagnosed via ultrasonography, computed tomography, or magnetic resonance imaging.

**Conclusion:**

Despite the lack of guidelines, intervention was necessary because the patient was anxious regarding the increase in the size of the swelling, which she felt had a negative psychosocial impact. Moreover, because the sizable venous aneurysm harbored a mural thrombus that increased the risk of embolization and pulmonary embolism, surgery was offered.

Indication for surgery includes pain, swelling, and cosmetic concerns. Conservative management of the condition is described in the literature.

## 1 Introduction

Aneurysm is a localized dilatation of an artery of at least 1.5 times the normal diameter that occurs when part of an artery wall weakens or is injured, allowing it to widen abnormally. In practice, arterial aneurysm is more common than venous aneurysm. This case report focused on the discussion, presentation, investigation, and treatment of internal jugular vein aneurysm.

## 2 Patient information

A 57-year-old Saudi woman, with no significant medical history, such as diabetes mellitus, hypertension, asthma, dyslipidemia, or other chronic condition, presented to Prince Sultan Military Hospital complaining of right-side neck swelling that started 3 years ago. The patient reported that the swelling enlarged with coughing and straining, but there was no pain, change in skin color, dysphagia, change in voice, neurological defect, shortness of breath, history of any trauma to the neck, surgical intervention, or any lump.

## 3 Clinical findings

Examination of the neck showed a round, compressible, nontender, nonpulsatile lump, measuring about 4 × 4 cm, on the right side on level 3; there was no change in the overlying skin.

Performance of the Valsalva maneuver by the patient enlarged the swelling. Upper and lower limb examination finding was normal. No other swelling was noted.

## 4 Timeline

### 4.1 Diagnostic assessment

Ultrasound examination showed a 5.1 × 3.8 × 1.4-cm cystic structure at the right side of the neck just above the level of the thyroid gland and anterior to the right internal jugular vein and the right internal carotid artery that contained fluid with internal echoes and with a thin wall. The structure was noncommunicating with the surrounding vascularity, which is consistent with second branchial cleft cyst; however, evaluation by magnetic resonance imaging (MRI) of the neck was advised to confirm the diagnosis and to rule out underlying fistula. The thyroid gland had a normal size and echogenicity with no lesions. No cervical lymph node enlargement was noted.

**Figure 1:**
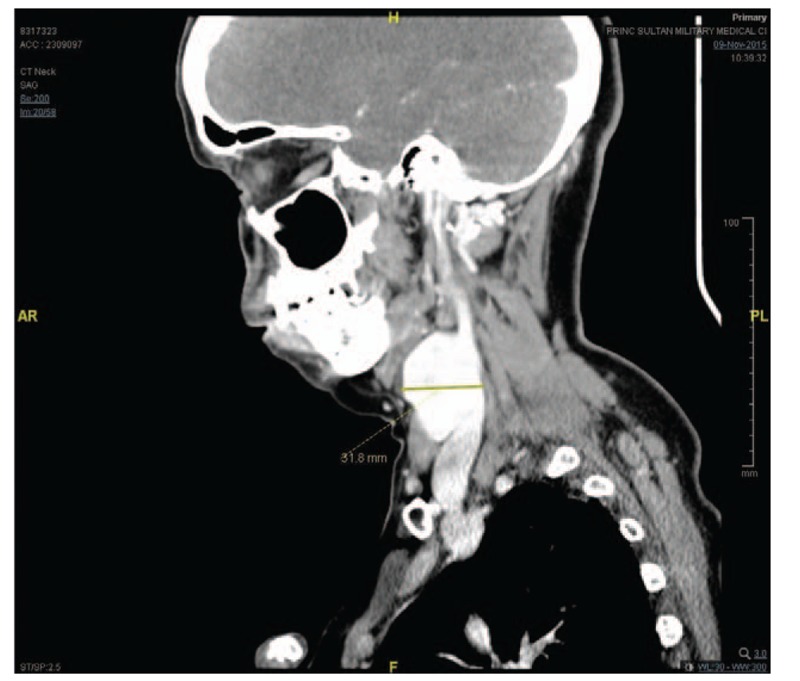
Right-sided neck saccular contrast-filled lesion visualized by contrast computed tomography.

**Figure 2:**
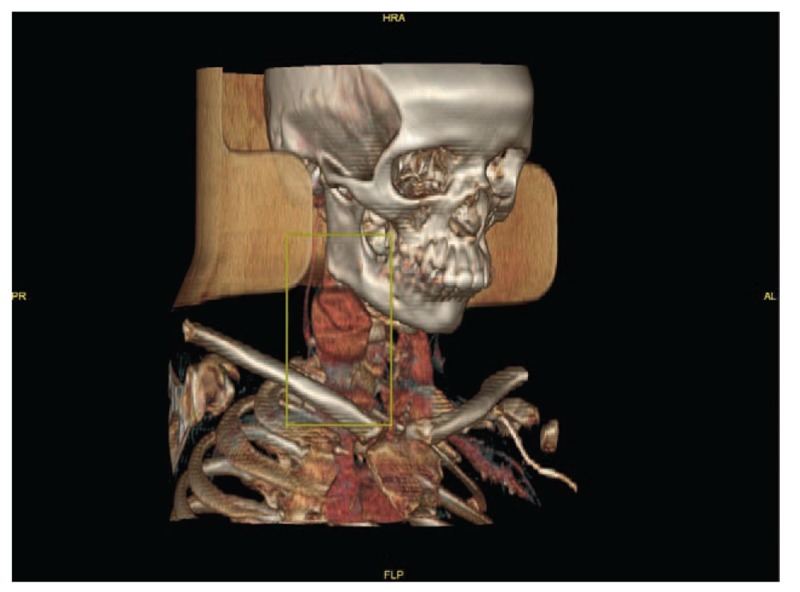
Three-dimensional view of the right-sided neck saccular contrast-filled lesion.

**Figure 3:**
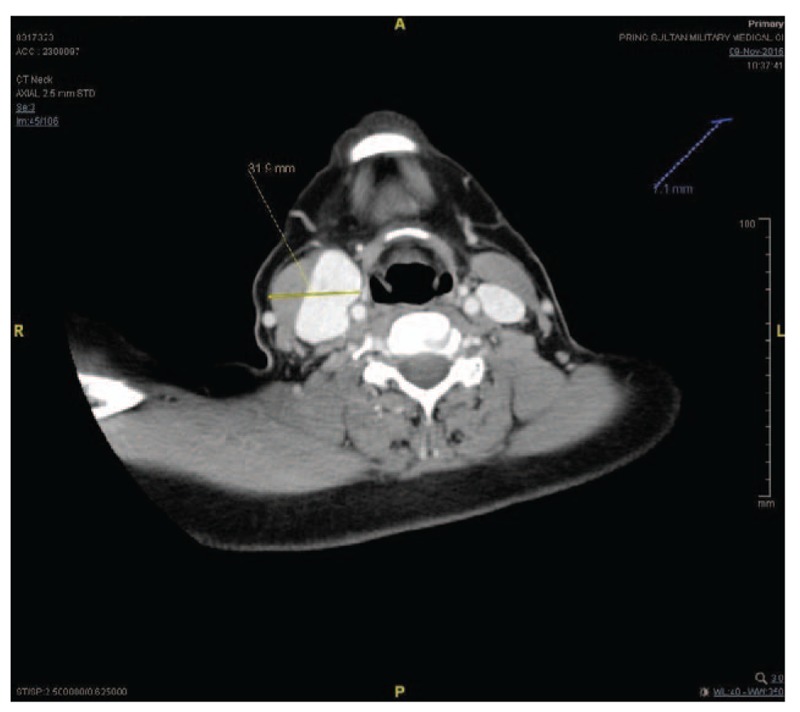
Computed tomography scan (axial view) showing an anterolateral filling defect, which could represent a thrombus.

Computed tomography (CT) scan showed a right-sided neck saccular contrast-filled lesion that was continuous with the lumen of the right internal jugular vein and measured about 4.5 × 3.0 cm in maximum diameter (Figs. 1 and 2). It displaced the right carotid vessels medially. Fig. 3 shows an anterolateral filling defect, which could represent a thrombus. A linear low-attenuation area is also seen at its posterior aspect, which could represent an intimal flap. Findings of the left carotid vessels and internal jugular vein were unremarkable. Although findings of the parotid and submandibular salivary glands were unremarkable, a subcentimetric cervical lymphadenopathy was noted. CT sections passing through the brain and upper chest showed no significant abnormality.

### 4.2 Therapeutic intervention

As the patient was anxious regarding the increase in the size of the swelling, which she felt had a negative psychosocial impact, and as the sizable venous aneurysm harbored a mural thrombus that increased the risk of embolization and pulmonary embolism, surgery was offered.

**Figure 4:**
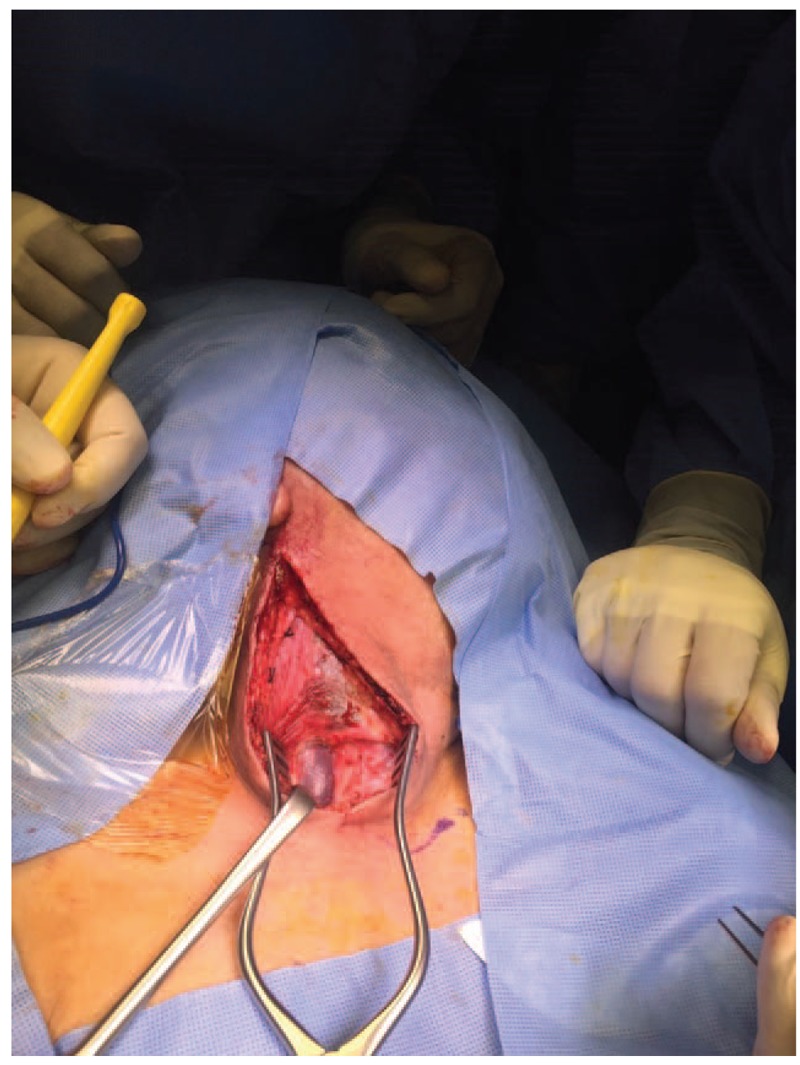
Incision along the anterior border of the sternocleidomastoid muscle.

**Figure 5:**
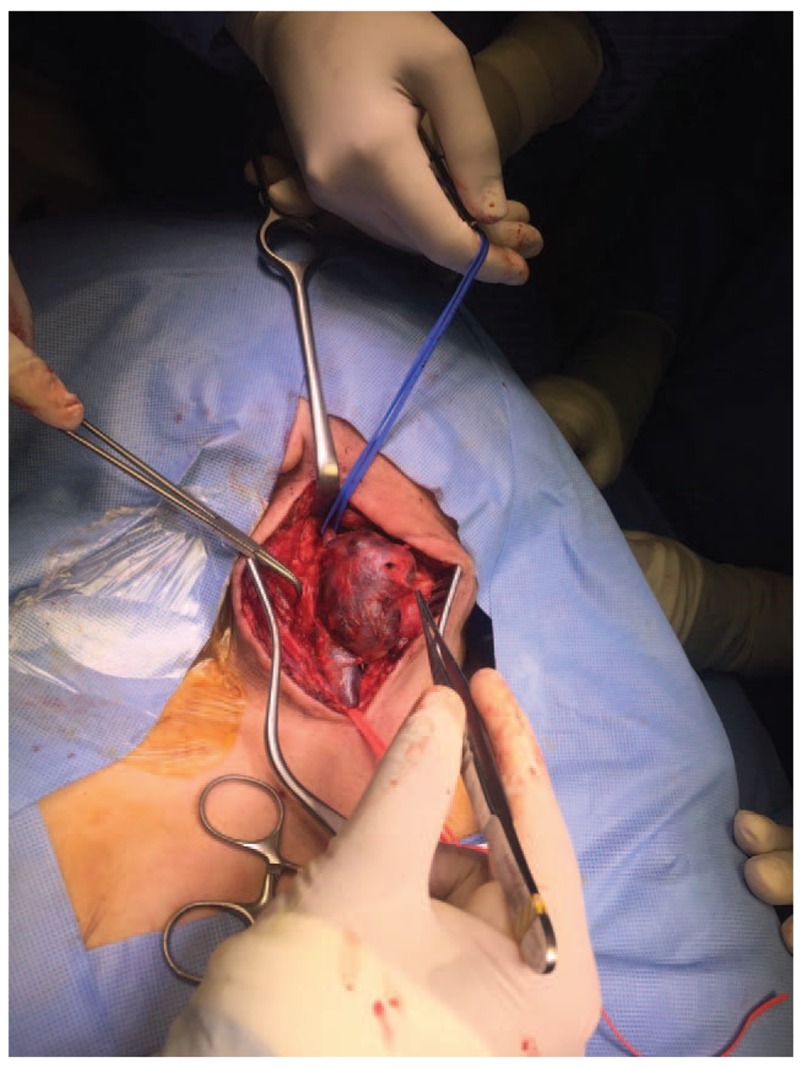
Separation of the internal jugular vein and the aneurysm from the muscle and the common carotid artery.

**Figure 6:**
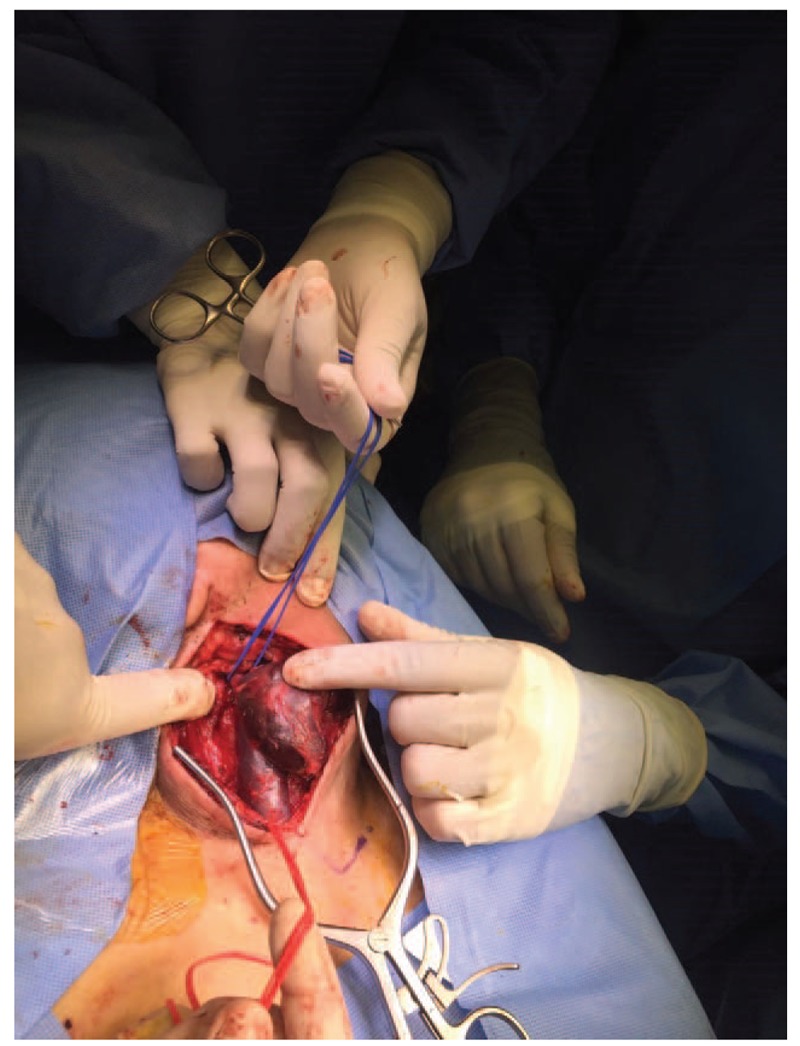
Proximal and distal control was achieved after the aneurysms were opened, evacuated, and excised.

**Figure 7:**
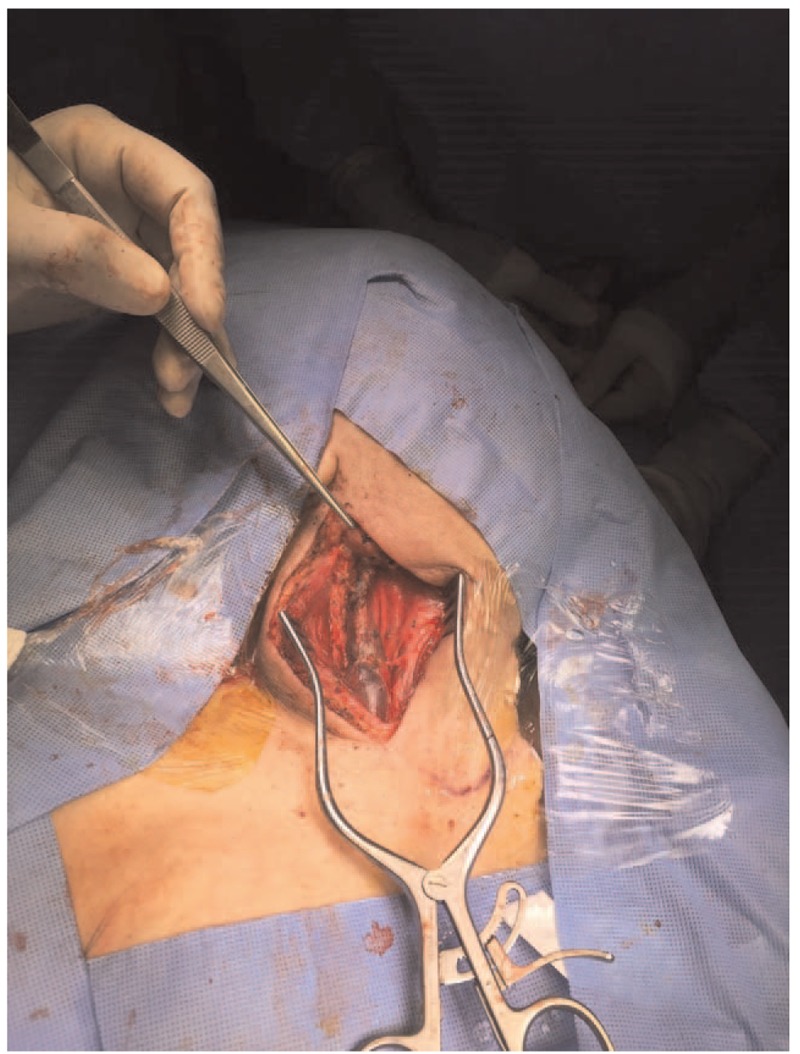
The wall of the internal jugular vein was closed using 5.0 Proline with continuous sutures.

Surgery was performed under general anesthesia through an incision along the anterior border of the sternocleidomastoid muscle (SCM) up to the carotid sheath (Fig. 4). The internal jugular vein was separated from the carotid artery and SCM muscle (Fig. 5). Then, the patient was anticoagulated with 5000 U of heparin (intravenous) and vascular clamps were applied proximal and distal to the aneurysm. Excisional venotomy was performed, and the mural thrombus was removed with partial wall excision (Fig. 6). Finally, the internal jugular vein was closed using 5.0 Proline suture (Fig. 7). Hemostasis was accomplished, and the specimen was sent for histopathological examination.

### 4.3 Follow-up and outcomes

The patient did well postoperatively, and 3 days later, enoxaparin 60 mg was subcutaneously administered and she was discharged. According to the patient, her condition has improved and she can function well again.

Follow-up duplex ultrasound performed 2 weeks and 2,4,6,12 months postintervention showed a patent internal jugular vein.

## 5 Discussion

Aneurysms are dilatations of the blood vessel wall that may include parts of the vessel or all its layers.^[1]^ They are more common in arteries, but venous aneurysms are also described in literature.^[2]^ This is a case of aneurysm of the internal jugular vein, which is responsible for the drainage of blood from the head, the brain, and the superficial parts of the face and neck and back to the heart.^[3]^

Venous aneurysms are rare and could present at any age, with no difference between the sexes or particular anatomical pattern (e.g., it can present as a mass in the neck that enlarges with coughing or straining).^[4]^ The majority of patients have asymptomatic mass,^[5,6]^ but symptoms such as pain and tenderness have been described.^[7]^ Differential diagnoses included hemangiomas, branchial and enterogenous cysts (thyroglossal cyst, dermoid cyst, branchial cyst, cystic hygroma), carotid body tumors, lymphoceles, laryngeal diverticula, cervical adenitis, thyroid mass, and persistent jugular lymphatic sac.^[6,8]^ Because of the rarity of this condition, we consulted different specialists, such as ENT and head and neck surgeons; furthermore, the lack of guidelines on the treatment of this condition posed a challenge. The diagnosis can be made through ultrasonography, CT, or MRI.^[6]^

Because of the rare incidence of venous aneurysms, treatment guidelines are not clearly established and the treatment strategies vary.^[9]^ Indication for surgery includes pain, swelling, and for cosmoses concerns. Moreover, conservative management are described in the literature.^[5,8,10]^
